# Monitoring phage infection and lysis of surface-immobilized bacteria by QCM-D

**DOI:** 10.1007/s00216-025-05803-5

**Published:** 2025-02-25

**Authors:** Bhanu K. Pothineni, René Probst, Dorothee Kiefer, Verena Dobretzberger, Ivan Barišić, Guido Grundmeier, Adrian Keller

**Affiliations:** 1 Technical and Macromolecular Chemistry, Paderborn University, Warburger Str. 100, 33098 Paderborn, Germany; 2 Institute of Biology, University of Hohenheim, 190h, Garbenstr. 30, 70599 Stuttgart, Germany; 3 Molecular Diagnostics, Center for Health and Bioresources, AIT Austrian Institute of Technology Gmbh, 1210 Vienna, Austria

**Keywords:** Bacteriophages, T7, *Escherichia coli*, Infection, Quartz crystal microbalance with dissipation monitoring, Phage therapy

## Abstract

While being a promising approach for the treatment of infections caused by drug-resistant, pathogenic bacteria, the clinical implementation of phage therapy still faces several challenges. One of these challenges lies in the high strain-specificity of most bacteriophages, which makes it necessary to screen large phage collections against the target pathogens in order to identify suitable candidates for the formulations of personalized therapeutic phage cocktails. In this work, we evaluate the potential of quartz crystal microbalance with dissipation monitoring (QCM-D) to identify and detect phage infection and subsequent lysis of bacteria immobilized on the surfaces of the QCM-D sensors. Using lytic *Escherichia coli* phage T7 as a model, we show that phage infection of *E. coli* cells results in various unique alterations in the behaviors of the frequency (Δ*f*) and dissipation (Δ*D*) signals, which are not observed during exposure of the *E. coli* strain to non-infectious *Bacillus subtilis* phage phi29 at similar concentration. To aid future phage screening campaigns, we furthermore identify a single measurement parameter, i.e., the spread between the different overtones of Δ*D*, that can be used to detect phage-induced lysis. For T7 infection of *E. coli*, this is achieved within 4 h after inoculation, including immobilization and growth of the bacteria on the sensor surface, as well as the completed phage propagation cycle. Given the commercial availability of highly automated multichannel systems and the fact that this approach does not require any sensor modifications, QCM-D has the potential to become a valuable tool for screening medium-sized phage collections against target pathogens.

**Graphical Abstract:**

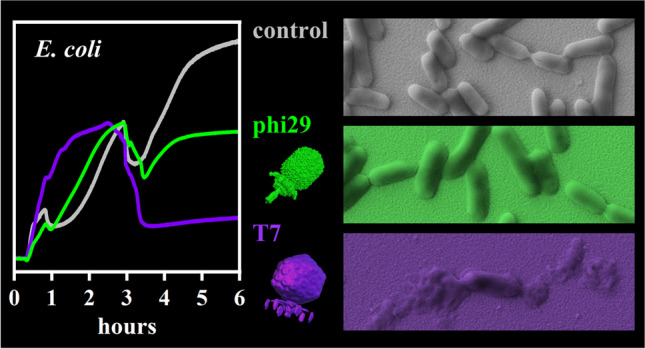

**Supplementary Information:**

The online version contains supplementary material available at 10.1007/s00216-025-05803-5.

## Introduction

Bacteriophages, also known as bacterial viruses, are minuscule particles that have the ability to infect bacteria and replicate exponentially by hijacking the host cell’s genetic and physiological machinery [1, 2]. Phages are omnipresent in nature, cohabiting with bacteria and sustaining their population through perpetual cycles of eradication. Consequently, new phages are continuously discovered in diverse environments ranging from the deep sea [3] to human wastewater [4]. While phages contribute to the spreading of bacterial virulence [5] and antibiotic resistance genes [6], they can also be utilized to treat antibiotic-resistant bacterial infections in what is called phage therapy [7]. However, while there is an increasing number of case studies reporting positive treatment outcomes under compassionate use, clinical trials so far had mixed success [7, 8]. The latter is rooted in a number of challenges that the clinical implementation of phage therapy faces. Chief among them is the high strain-specificity of many phages, which requires the preparation of cocktails of different strain-specific phages that can effectively treat a large number of clinical isolates of the target pathogen [7–9]. To achieve this, large collections of hundreds of phages need to be screened [8, 9]. While several methods have been developed or adapted for this purpose, ranging from well-established plaque assays [10] to more sophisticated techniques such as qPCR [11], surface plasmon resonance [12], atomic force microscopy [13], and several fluorescence-based assays [14, 15], all these approaches have their own disadvantages and limitations, in particular regarding response time, universality, ease of use, and throughput [8]. Furthermore, phage therapy is considered a promising strategy against biofilm-related infections that are particularly difficult to treat [16]. Most high-throughput screening techniques, however, are compatible only with planktonic cells. Consequently, there is a growing demand for surface-sensitive analytical techniques that enable the efficient screening of phages against bacterial biofilms.

Quartz crystal microbalance with dissipation monitoring (QCM-D) is a label-free technique that detects changes in the mass and viscoelasticity of a thin adsorbate film on the surface of a piezoelectric quartz sensor in situ and in real time. An AC voltage is applied across the sensor, which in response undergoes a shear oscillation at its resonance frequency in the MHz range. Adsorption or desorption events on the sensor surface result in shifts in the sensor’s resonance frequency Δ*f* and energy dissipation Δ*D*, which can be translated into changes in mass and viscoelastic properties, respectively, by the application of appropriate models [17]. Because of its exceptional mass sensitivity [18] down to a few ng/cm^2^ and the commercial availability of highly automated multichannel systems [19], QCM-D has become a powerful method for the screening of biomolecular and cellular interactions. As such, it is employed in various biomedical research fields such as drug discovery [20, 21], drug delivery [22, 23], amyloid aggregation [24, 25], bacterial adhesion [26, 27], and biofilm formation [28, 29].

In this work, we evaluate the potential of QCM-D to monitor phage infections of bacteria in real time. To this end, *Escherichia* *coli* cells are adsorbed on the gold surface of the QCM-D sensor and subsequently exposed to infectious bacteriophage T7 or non-infectious phage phi29. T7 was specifically selected because of its short lytic life cycle of only 17 min [30]. We demonstrate that this approach is able to distinguish infectious from non-infectious phages within 4 h. We also show that phage-induced lysis can be detected using only a single measurement parameter, i.e., the spread between the different overtones of the Δ*D* traces, which substantially reduces the complexity of the evaluation of the sensor response and will aid future phage screening campaigns.

## Materials and methods

### Materials

*E. coli* B (DSM 613) and T7 phage were provided by the University of Hohenheim. Bacillus phage phi29 HER 243 (DSM 5546) and its designated propagation strain *B. subtilis* HER 1243 (DSM 5547) were purchased from DSMZ, Germany. LB medium was purchased from MP Biomedicals, Germany; eosin methylene blue (EMB) agar (Oxoid) from ThermoFischer Scientific, UK; and chloroform from Macron Fine Chemicals, USA. Peptone extract and agar powder food grade were purchased from PanReac AppliChem, Germany. Phosphate-buffered saline (PBS), MacConkey agar, and glucose were purchased from Sigma-Aldrich, Germany. NaCl (≥ 99.5%), yeast extract, and tryptone were purchased from Merck, Germany. Agar powder, MgSO_4_, KH_2_PO_4_, and molecular biology-grade water were purchased from VWR, Germany. Na_2_HPO_4_ (98%) and aniline blue dye were purchased from Thermo Scientific, Germany. Hellmanex was purchased from Hellma GmbH, Germany; ethanol p.a. from Berkel AHK GmbH & Co. KG, Germany; and HPLC-grade water from Roth, Germany. NH_4_OH (35%) and H_2_O_2_ (25%) were purchased from Stockmeier Chemie, Germany.

### Bacteria and phage culture

*E. coli* B was cultured in LB medium at 37 °C with constant shaking (222DS, Labnet, USA) overnight. The next day, 3 mL of fresh LB medium was inoculated with 100 µL of the overnight culture of bacteria and was grown until it reached an OD600 value of 0.6–0.7. The bacterial culture at this point was infected with 100 µL of T7 phage solution (m.o.i. ≈5) and incubated at 37 °C with constant shaking. The bacterial cell lysis and subsequent cell death were verified by a drop in the OD value to 0.05 or less. After this, 150 µL of chloroform was added to the medium and the solution was vortexed briefly for 10 s, after which it was incubated on ice for 5 min. Then, the supernatant was transferred to a new 15-mL centrifuge tube and centrifuged at 8800 rcf for 5 min. The supernatant containing the phages was transferred to a fresh tube. The phage concentration was quantified using a water blue double overlay plaque assay as previously described [31]. To this end, agar plates were prepared by dissolving 4 g of peptone extract, 0.7 g of NaCl, and 3 g of agar in 183 mL of molecular biology-grade water. This mixture was autoclaved at a temperature of 121 °C for 15 min. After letting the solution cool to 80 °C, 12 mL of a 25% glucose solution was introduced, followed by the addition of 2.7 mL of 0.5 M Na_2_HPO_4_ and 1.6 mL of aniline blue dye (stock solution 1% in sterile, molecular biology-grade water). Subsequently, the solution was poured into Petri dishes and allowed to solidify. In parallel, a 0.7% water agar was prepared and maintained at a temperature of 47 °C until further use. The phages were serially diluted tenfold in autoclaved phosphate buffer (38.3 mM Na_2_HPO_4_ · 2 H_2_O, 68.4 mM NaCl, 22 mM KH_2_PO_4_), supplemented with 1 mM MgSO_4_. A mixture of 300 µL of a bacterial liquid culture of OD ~ 1.0 and 100 µL of phage solution was combined with 4 mL of the water agar and briefly vortexed. The resulting mixture was then poured onto the pre-warmed water blue bottom agar plates. The agar was left to solidify, and the plates were incubated at a temperature of 37 °C overnight. The following day, the plaques were counted to determine the phage titer.

To verify the purity of the *E. coli* culture, the bacteria were quadrant streaked on MacConkey and EMB agar, which are selective media for enteric bacteria. MacConkey agar was prepared by dissolving 5 g of powder in 100 mL of molecular biology-grade water. EMB agar was prepared by dissolving 3.75 g powder in 100 mL of molecular biology-grade water. Both agars were autoclaved at 121 °C for 15 min.

*B. subtilis* was cultivated and grown to exponential phase as described for *E. coli*. For the confirmation of the phi29 phage solution purity, *B. subtilis* cells were centrifuged for 10 min at 4100 rcf and resuspended in low salt LB (1% Bacto-Tryptone, 0.5% yeast extract, 0.5% NaCl) to an OD of ~ 10. Subsequently, 40 µL of this suspension was transferred to 5-mL tubes and infected with 5 µL of four different phage dilutions (100-fold). To the mixture, 4 mL top agar was added (low salt LB with 0.7% agar food grade, pre-warmed at 50 °C), inverted several times and poured onto bottom plates (low salt LB with 1.5% agar, pre-warmed at 37 °C). After overnight incubation at 37 °C, distinct plaques should be visible with at least one phage dilution. When uniform morphology and purity were confirmed, single plaques were used to infect an overnight culture diluted to an OD ~ 0.2 (1 plaque per 100 mL) and kept for phage propagation at 37 °C with shaking overnight. Next day, the culture was centrifuged for 15 min at 4800 rcf to separate the supernatant containing the phages from the bacteria pellet. Additionally, a PES 0.22-µm membrane filter unit (Merck, Germany) was used to remove residual debris and bacterial cells. Finally, the titer was determined via layer plating as described above using a tenfold phage dilution series.

### Atomic force microscopy

Gold-coated QCM-D sensors (5 MHz 14 mm Cr/Au, Quartz Pro, Sweden) from three different batches were rinsed with ethanol and HPLC-grade water and dried with argon. The cleaned samples were imaged in air using a Bruker Dimension Icon in ScanAsyst Peak-Force tapping mode with ScanAsyst air cantilevers (Bruker, Germany). Images were recorded at 1024 × 1024 pixels with a scan size of 5 µm^2^. Gwyddion [32] was used to process the images and calculate the rms surface roughness *S*_q_.

### Preparation of QCM-D flow cells and sensors

The flow cells and the inlet pipes of the QCM-D system (E4, Biolin Scientific, Sweden) were incubated in 2% Hellmanex for 2 h. Afterwards, the flow cells were rinsed with 99% ethanol and HPLC-grade water, and dried with ultrapure argon. The gold-coated QCM-D sensors were cleaned in RCA-1 solution (1:1:5 in volume 35% NH_4_OH, 25% H_2_O_2_, H_2_O) at 75 °C for 1 min. After cleaning, the sensors were rinsed with HPLC grade water and dried with argon.

### QCM-D experiments

All buffers and solutions were autoclaved prior to the experiments. *E. coli* B was cultured overnight in LB medium at 37 °C with constant shaking. The next day, 20 mL of fresh LB medium was inoculated with 500 µL of the overnight *E. coli* B culture. The bacteria were grown to an OD600 value of 0.6 (mid log phase culture). The T7 phages were diluted in LB medium to three different concentrations of 8 × 10^7^ PFU/mL, 2 × 10^6^ PFU/mL, and 4 × 10^5^ PFU/mL, respectively. QCM-D measurements were conducted at 37 °C, either in static mode or with a constant flow rate of 30 µL/min. PBS was first pumped into the flow cell for 20 min to obtain a constant baseline, after which bacteria in LB medium were pumped in for 30 min to adsorb to the sensor surface. Afterwards, the flow cell was flushed for 15 min with bacteria-free medium to remove unattached bacteria. Then the flow was stopped, and the bacteria were left to grow under static conditions for 2 h. Phages in LB medium were then pumped in for 20 min, followed by another medium flush for 15 min to remove excess phages not attached to the bacteria. The flow was then stopped again, and the sample incubated for 3 h under static conditions. In order to evaluate the behavior of the adsorbed bacteria in the absence of phages, one control used phage-free LB medium instead of phage-containing medium. To evaluate phage adsorption on the gold surface of the sensor, an additional control used bacteria-free medium instead of bacteria-containing medium.

The QCM-D data was processed using D-find software (Biolin Scientific, Sweden) and plotted using Origin Pro 2021 (OriginLab, USA).

### Scanning electron microscopy

After the QCM-D experiments, the sensors were incubated in 2.5% glutaraldehyde in PBS overnight. Subsequently, the sensors were dehydrated by incubating them sequentially in ethanol dilutions of 20%, 40%, 60%, 80%, and 100% for 10 min each. After the last incubation step, the sensors were dried in air and their surfaces sputter-coated (SCD 500, Leica Microsystems, Germany) with a 3-nm-thick gold alloy (80% Au + 20% Pd). Finally, the sensors were examined using a NEON 40 SEM (Zeiss, Germany) at various magnifications (1 k, 3 k, 10 k, 15 k, 25 k, and 40 k) with a 5-kV electron beam.

## Results and discussion

### *E. coli* adsorption, adhesion, and growth on the QCM-D sensor surface

**Fig. 1:**
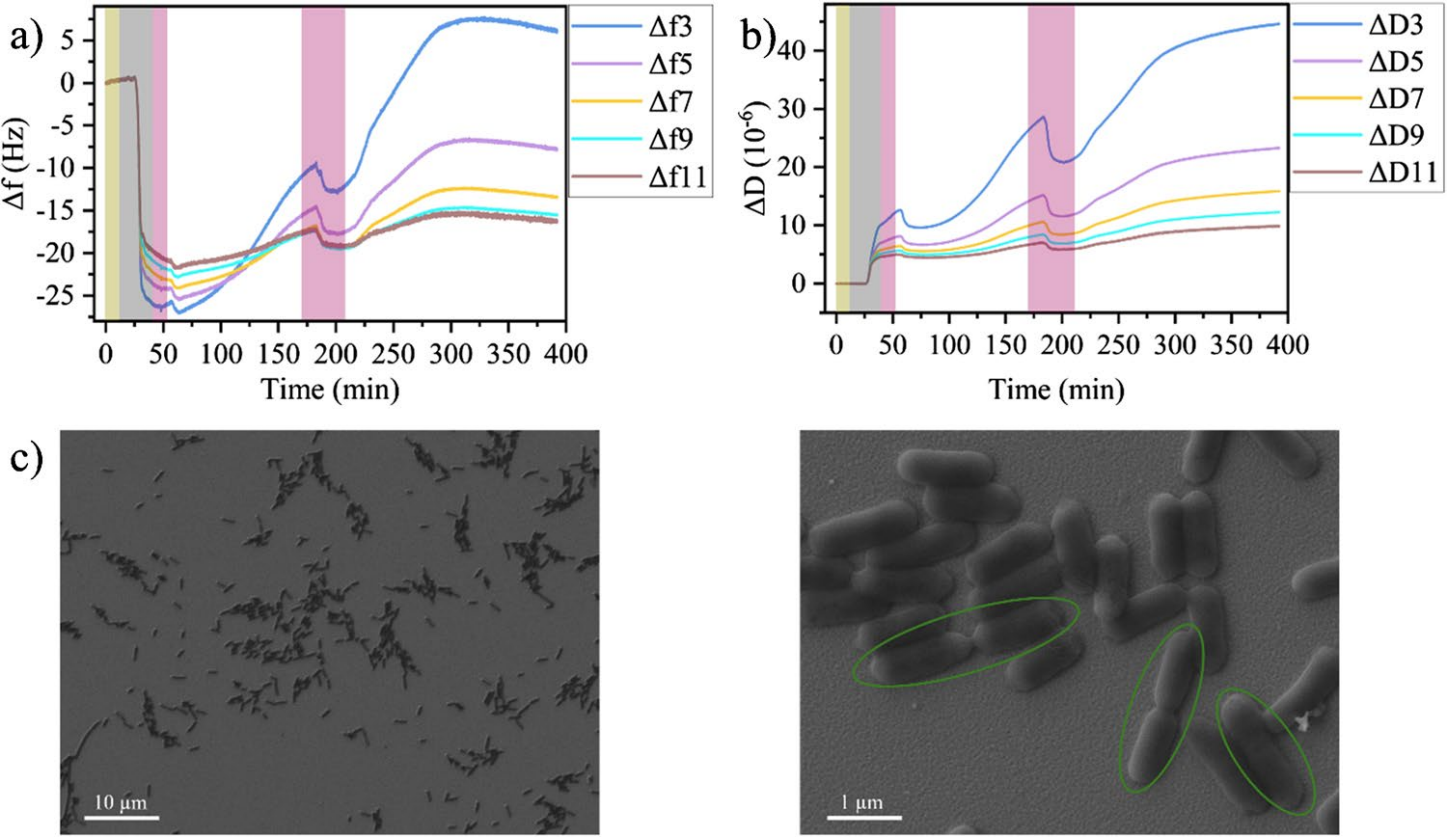
Change in frequency Δ*f* (**a**) and dissipation Δ*D* (**b**) during *E. coli* adsorption and growth on the QCM-D sensor surface. Shaded regions indicate the injection of different media: yellow—PBS, gray—bacteria in medium, pink—medium. **c** SEM images of the sensor surface after the experiment at two different magnifications. Dividing bacteria are highlighted. Overtones in **a** and **b** are indicated by identical colors

Before investigating the effects of T7 infection by QCM-D, Fig. 1a and b evaluate the QCM-D response of *E. coli* adsorption and growth on the QCM-D sensor in the absence of any phages for the overtones 3 to 11. Injection of bacteria-containing LB medium results in a rapid decrease in Δ*f* for all overtones, which corresponds to an increase in mass and thus is indicative of the adsorption of bacteria on the sensor surface. At the same time, Δ*D* shows a rapid increase, indicating that the system becomes more viscoelastic due to adsorbing and adhering bacteria. Both Δ*f* and Δ*D* saturate rather quickly, at which point their overtones start separating from each other. Flushing of the flow cell with bacteria-free medium results only in minor changes in the Δ*f* and Δ*D* traces, indicating that the bacteria stably adhere to the sensor surface. After flushing, the pump was stopped, and the behavior of the adhering bacteria was monitored under static conditions. Over the following 2 h, both Δ*f* and Δ*D* increase in value, which is accompanied by increased spreading between the overtones of Δ*D*. The increase in Δ*D* as well as the large spread between its individual overtones is consistent with bacteria growing on the surface. A larger number of adhering bacteria leads to more efficient energy dissipation due to their intrinsic viscoelasticity and the enhanced coupling to the liquid phase. At the same time, the effective thickness of the bacterial film increases with the number of bacteria, which accounts for the increased spread between overtones. This is because the penetration depth of the acoustic shear wave into the medium decreases with increasing overtone number [33]. For the third overtone, the penetration depth into an aqueous medium is about 140 nm, which is considerably smaller than the diameter of an *E. coli* cell [34]. The 11th overtone, however, has an even smaller penetration depth of only about 70 nm [33]. Therefore, while the lower overtones sense a certain fraction of the more liquid-like bacterial cytoplasm, the higher overtones are more sensitive toward the stiffer cell envelope close to the sensor surface. Somewhat surprisingly, Δ*f* increases as well in this regime. At first glance, this indicates the desorption of bacteria instead of bacterial growth. Nevertheless, such positive shifts of Δ*f* are frequently observed during bacterial adhesion and biofilm formation on the sensor surface, which follows a coupled resonance model instead of conventional Saurbrey theory [28, 35–37]. In this context, it was shown that Δ*f* is not a reliable measure of the number of bacterial cells adhering to the sensor surface [38], so that detailed analyses of their behavior ought to be based on Δ*D* [39, 40].

Flushing the flow cell once more with medium about 170 min after the start of the experiment leads to a small decrease in both Δ*f* and Δ*D*. This is indicative of the desorption of loosely bound bacteria from the sensor surface and a simultaneously enhanced adhesion of the remaining bacteria. Immediately after flushing, both Δ*f* and Δ*D* increase again until they saturate about 100 min later. In this phase, the spread between the individual overtones increases as well. These behaviors of the Δ*f* and Δ*D* traces are thus consistent with the resumed growth of bacteria firmly attached to the sensor surface, which continues until the medium in the flow cell (volume ~ 40 µL) is depleted of nutrients. Bacterial growth was further verified by SEM imaging of the sensor surface after the end of the QCM-D measurement. Several stages of dividing bacteria (see Fig. 1c and Figs. S1 and S2) can be identified, which proves that bacterial growth is resumed after addition of fresh medium. The SEM images in Figs. S2 and S3 further reveal several long filamentous bacterial cells. MacConkey and EMB agar cultures (see Fig. S4) of these cells show uniform and coherent colonies. This reveals that the filamentous cells observed in the SEM are indeed *E. coli* and no contaminating cells. Filamentation of *E. coli* and other bacteria is a common phenomenon [41–44]. While occurring naturally at a low rate, filamentation is induced under stress conditions, including starvation, temperature shock, and antibiotics exposure [45]. However, surface immobilization and shear flows have also been identified as factors that may stimulate filamentation of *E. coli* [46]. In our experiments, the bacteria are immobilized on the sensor surface, subjected repeatedly to shear flows, and grown under static conditions with limited nutrient availability, all while being exposed to acoustic surface waves of 5 MHz. The stress resulting from those conditions may induce filamentation of some of the immobilized cells indeed. This filamentation may then further lead to an additional increase in dissipation.

A general issue in QCM-D is the strong dependence of the measurement on the individual sensor employed, with technically identical sensors often having rather different sensitivities and responses. In these experiments, the strongest deviations between measurements are observed in the Δ*f* traces, which in some cases show positive frequency shifts during bacterial adhesion and growth, while in other cases, negative shifts are observed (see Figs. 1, S5, and S6). The Δ*D* curves, however, follow rather similar trends, in further support of Δ*D* being the more relevant parameter for evaluating bacterial adhesion and growth. Here, the most notable differences are observed after the second injection of medium, i.e., in the second growth stage. While flushing with medium in all experiments leads to a small, transient drop in Δ*D* that is followed by an increase with subsequent saturation, the overall magnitude of the increase and the saturation level vary. The SEM images shown in Figs. 1 and S5 suggest that these differences result from a variable, heterogeneous bacterial coverage and/or the formation of microcolonies on the sensor surface, which most likely are caused by varying surface properties of the individual gold electrodes. Even though AFM reveals rather similar surface topographies (see Fig. S7), surface inhomogeneities over larger length scales may lead to locally different bacterial adhesion and growth.

### T7 phage infection of *E. coli* on the QCM-D sensor surface

**Fig. 2:**
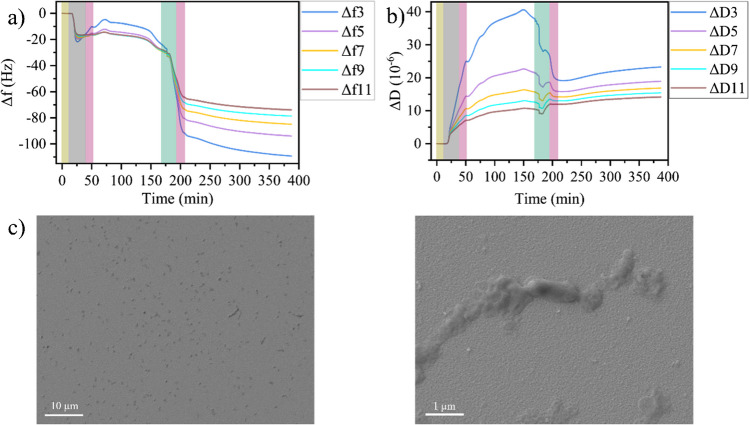
Change in frequency Δ*f* (**a**) and dissipation Δ*D* (**b**) during *E. coli* adsorption, growth, and T7 infection on the QCM-D sensor surface. Shaded regions indicate the injection of different media: yellow—PBS, gray—bacteria in medium, pink—medium, green—T7 phages in medium (8 × 10^7^ PFU/mL). **c** SEM images of the sensor surface after the experiment at two different magnifications. Overtones in **a** and **b** are indicated by identical colors

To follow phage infection by QCM-D, T7 phages in medium were injected during the second flushing step after ca. 165 min (see Fig. 2a, b). The phages at a concentration of ca. 8 × 10^7^ PFU/mL were pumped through the flow cell for 30 min to facilitate their binding to the bacteria. Afterwards, the flow cell was flushed again with phage-free medium to remove unbound phages. As soon as the flow cell is flooded with phages, both Δ*f* and Δ*D* show a rapid decrease that exceeds that observed for flushing with phage-free medium. For instance, for the third overtone, Δ*f* drops by about − 60 Hz when phages are injected, but by less than − 5 Hz for flushing with phage-free medium in the bacterial control without phages (see Fig. 1a). This indicates the rapid adsorption of phages on the bacteria and the free sensor surface. Bacteria-free control experiments with the sensors exposed to phages only reveal a much larger drop in Δ*f* due to phage adsorption on the free gold surface (see Fig. S8). This drop in Δ*f*, however, is accompanied by a large *increase* in Δ*D*, whereas in the presence of bacteria on the sensor surface, Δ*D decreases* upon phage injection (see Fig. 2b). In addition, this decrease is about twice as large as the one observed in Fig. 1b for the flushing with phage-free buffer. This verifies that the behavior observed in Fig. 2a and b does not result from simple, non-specific phage adsorption on the QCM-D sensor, but additionally involves specific interactions between phages and bacteria. After flushing with phage-free medium, the Δ*f* and Δ*D* traces both rapidly saturate before entering an extended regime, in which Δ*f* shows a slight but continuous decrease while Δ*D* increases correspondingly (see Fig. 2a, b). The sensor response in this regime is very different from that observed in Fig. 1a and b and does not show any indication of bacterial growth. Notably, both the absolute Δ*D* values and the spreading of the individual overtones at the end of this regime are much lower than those observed in Fig. 1b, indicating a more rigid adsorbate film.

These observations are consistent with the phage-induced lysis of the bacterial cells on the sensor surface. Upon lysis, the cell envelope ruptures, the cytoplasm is released into solution, and the empty envelope collapses onto the surface. This leads to a stiffer and thinner adsorbate film, which accounts for the decrease in Δ*D* and the reduced spread of its overtones. While the large decrease in Δ*f* during phage injection can be attributed to phage adsorption, the subsequent spread between overtones is now consistent with the presence of a viscous adsorbate layer that consists mostly of cellular debris. However, the slight, continuous increase in Δ*D* over the following 3 h may indicate that also some bacteria survived on the sensor surface and now resume to grow. Indeed, while the SEM images in Fig. 2c clearly show that the majority of bacteria on the sensor surface have been lysed, a few intact cells can be identified among the disintegrated ones. Furthermore, we would like to point out that cell lysis produces more reproducible sensor responses than cell growth (see Figs. 2 and S9), which underscores the potential of QCM-D to aid in the screening of phage libraries.

**Fig. 3:**
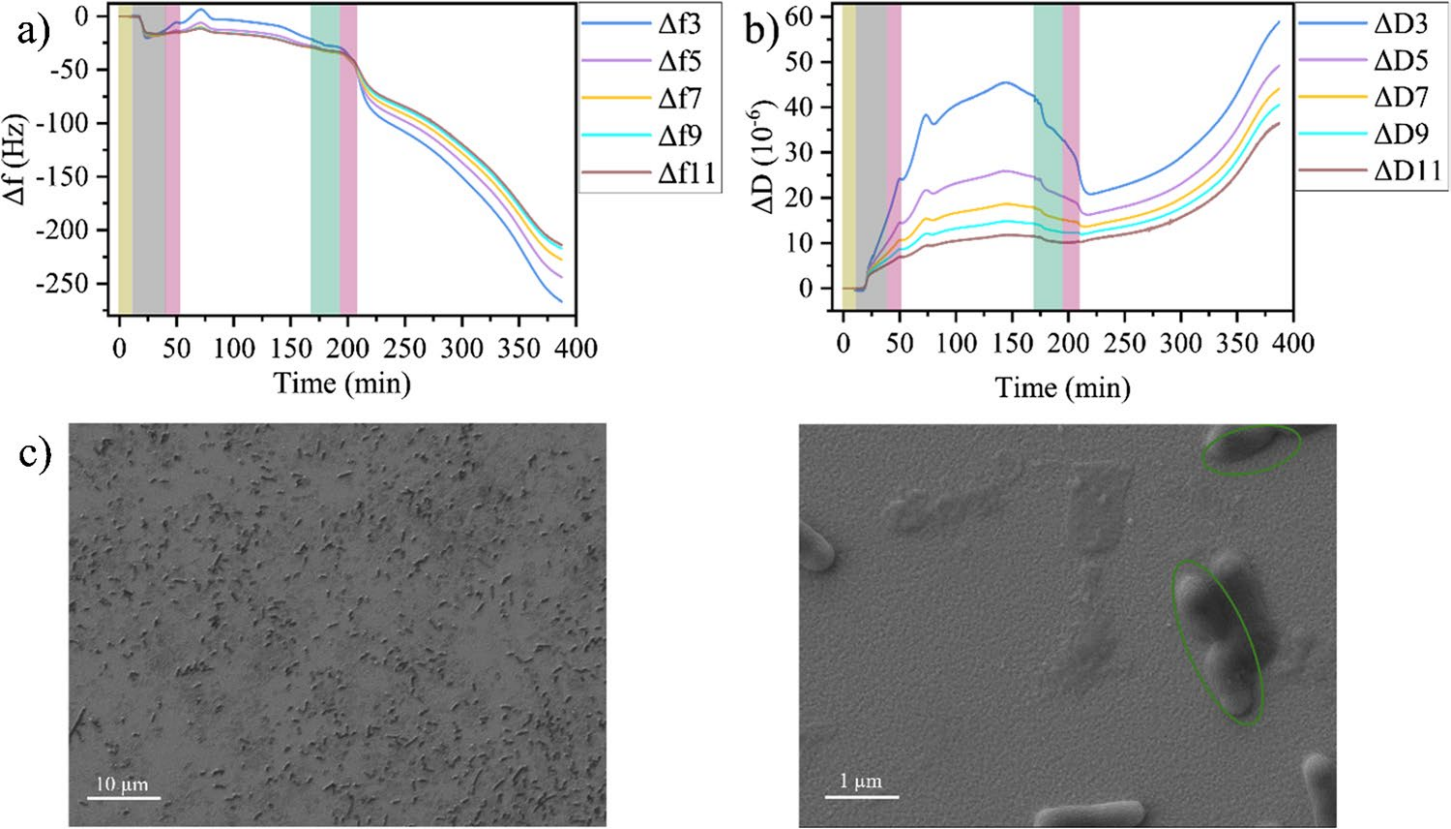
Change in frequency Δ*f* (**a**) and dissipation Δ*D* (**b**) during *E. coli* adsorption, growth, and T7 infection on the QCM-D sensor surface. Shaded regions indicate the injection of different media: yellow—PBS, gray—bacteria in medium, pink—medium, green—T7 phages in medium (2 × 10^6^ PFU/mL). **c** SEM images of the sensor surface after the experiment at two different magnifications. Dividing bacteria are highlighted. Overtones in **a** and **b** are indicated by identical colors

Due to the short lytic cycle of T7 of less than 20 min and the comparably high phage concentration of 8 × 10^7^ PFU/mL used in the above experiments, lysis of most bacteria will be completed before the flushing with phage-free medium is finished. In order to assess the robustness of the detection scheme, we next lowered the phage concentration to 2 × 10^6^ PFU/mL. This will lead to more bacteria surviving the initial phage exposure, while the subsequent flushing will remove free phages from the flow cell so that the surviving cells have a much smaller chance of getting infected. As can be seen in Fig. 3a and b, the overall behavior of the Δ*f* and Δ*D* traces up until the final flushing step after phage injection is very similar to that observed at the higher phage concentration in Fig. 2a and b. Only the dynamics of both traces during phage injection appear somewhat slowed down or even delayed, while the overall magnitudes of the observed decrease in Δ*f* and Δ*D* are rather similar to the previous experiment with higher phage titers. This is particularly interesting as the changes in ∆*f* and ∆*D* values upon phage adsorption on the sensor surface in the absence of bacteria are considerably reduced at the lower phage concentration (see Figs. S8 and S10). This is a clear indication that the observed changes are indeed related to phage infection and lysis of the adhering bacteria.

The most obvious differences in the Δ*f* and Δ*D* traces of the two phage concentrations are visible in the final regime after flushing. At the lower phage concentration, Δ*f* and Δ*D* immediately start to decrease and increase, respectively, and continue to do so at a much higher rate than for the higher phage concentration. This indicates that as expected, more bacteria survived the phage exposure at this lower concentration and resumed growth afterwards. The larger number of surviving bacteria is also supported by the SEM images, which revealed many intact bacteria on the sensor surface (see Fig. 3c). Furthermore, dividing bacteria are observed as well (see Fig. 3c), supporting our assumption of resumed growth. It should be stressed here that these experiments were conducted under static conditions in ~ 40 µL of medium, i.e., under limited nutrient availability. Lysis of bacterial cells releases additional nutrients into the environment, which in the absence of a flow remain available to the surviving cells and can promote their growth.

At an even lower T7 concentration of 4 × 10^5^ PFU/mL, a rather similar behavior is observed as at a concentration of 2 × 10^6^ PFU/mL (see Fig. S11). In particular, the Δ*D* traces in both cases exhibit a slow yet notable increase about 100 min after the second flushing step, indicative of the resumed growth of the surviving bacteria on the sensor surface. This demonstrates that bacterial lysis can be detected reliably in a certain time window also at comparably low phage concentrations.

### Exposure to *B. subtilis* phage phi29

**Fig. 4:**
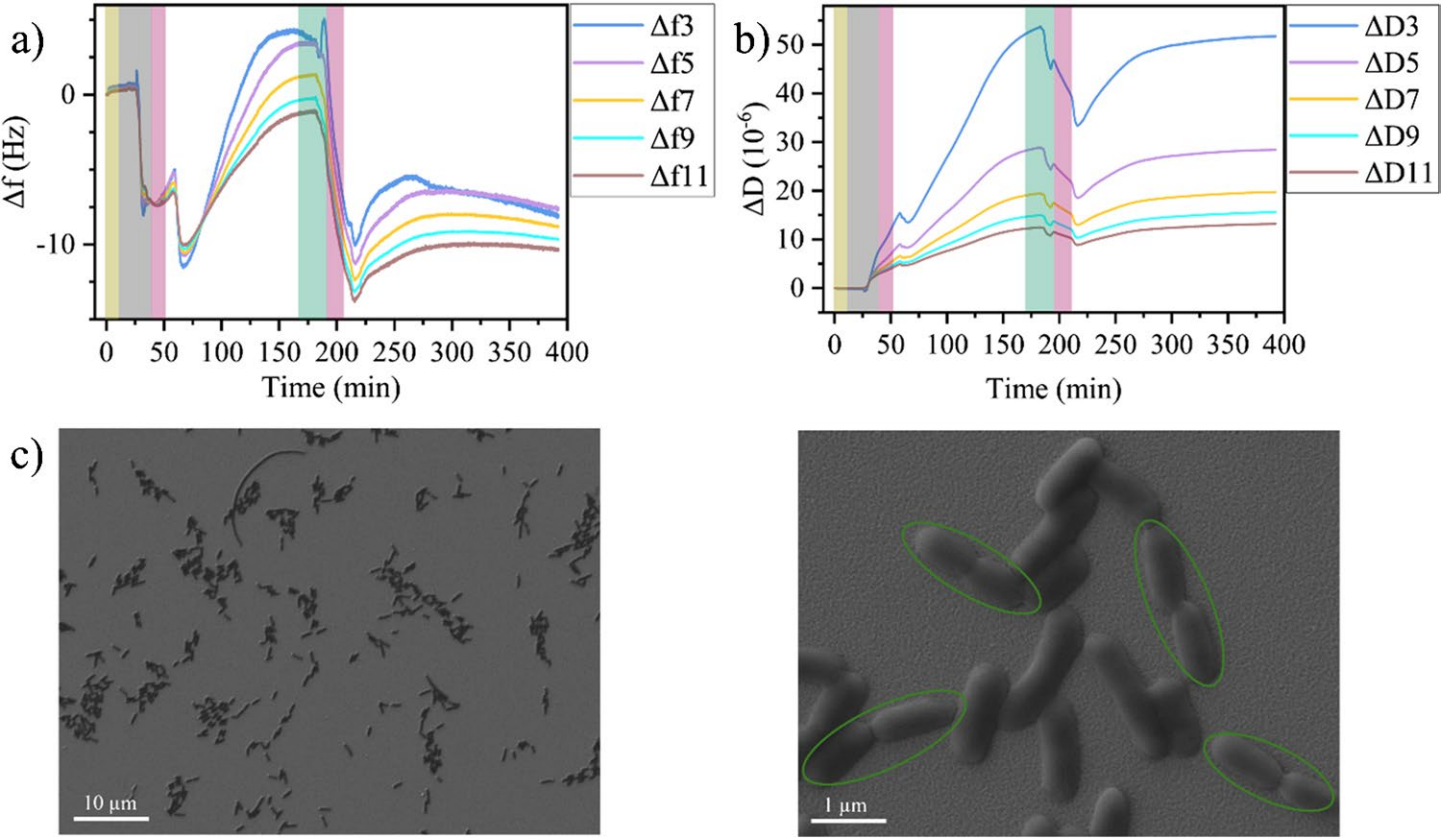
Change in frequency Δ*f* (**a**) and dissipation Δ*D* (**b**) during *E. coli* adsorption, growth, and phi29 exposure on the QCM-D sensor surface. Shaded regions indicate the injection of different media: yellow—PBS, gray—bacteria in medium, pink—medium, green—phi29 phages in medium (2 × 10^8^ PFU/mL). **c** SEM images of the sensor surface after the experiment at two different magnifications. Dividing bacteria are highlighted. Overtones in **a** and **b** are indicated by identical colors

Finally, we evaluated the QCM-D response to the exposure of the immobilized *E. coli* cells to a non-infecting phage. We chose bacteriophage phi29, which has similar dimensions as T7 but only infects *Bacillus subtilis* [47]. Figure 4 shows that injection of phi29 at 2 × 10^8^ PFU/mL results in a rapid drop in both Δ*f* and Δ*D*. After flushing with medium, however, both traces quickly recover and increase again until they saturate at about 250 min. The behavior in this regime thus resembles the late-stage growth phase observed in the absence of any phages in Figs. 1, S5, and S6 and indicates the resumed growth of the bacteria after phi29 injection. This is further verified by SEM (Fig. 4c), which reveals live and dividing *E. coli* bacteria on the sensor surface.

### Detecting phage-induced lysis by a single measurement parameter

**Fig. 5:**
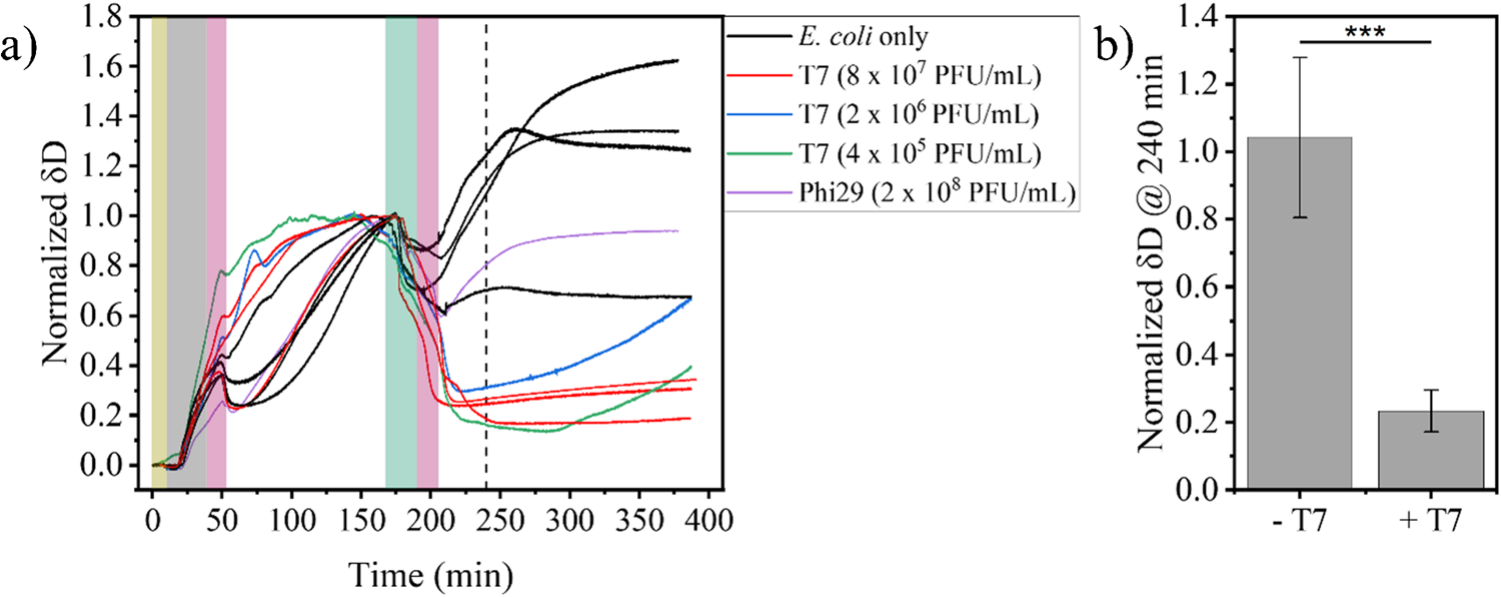
**a** Normalized δ*D* for the experiments shown in Figs. 1, 2, 3, 4, S5, S6, S9, and S11. Shaded regions indicate the injection of different media: yellow—PBS, gray—bacteria in medium, pink—medium, green—phages or medium (*E. coli* only). **b** Comparison of the normalized δ*D* at 240 min (indicated by the dashed vertical line in **a**) averaged over all *E. coli* only experiments (− T7) and T7 experiments (all concentrations, + T7). Values are given as the mean ± standard deviation. Statistical significance was determined by two-tailed *t*-test and is indicated as ***p < 0.001

Our experiments demonstrate the ability of QCM-D to monitor phage infections of bacteria immobilized on the sensor surface in real time. However, the associated changes in the different overtones of Δ*f* and Δ*D* are rather complex and depend on the individual sensor. This may render the verification of a successful infection and lysis of the target bacterium in a screening campaign with a large phage collection a difficult task. Therefore, we sought to identify a single measurement parameter whose time dependence during the experiments provides a clear and reliable indication whether phage-induced lysis of the target bacterium occurs. Based on the measurements shown in Figs. 1, 2, 3, and 4, the spread of the Δ*D* overtones appeared to be the most promising candidate for this purpose. To quantify the total spread, we simply calculated the difference between the absolute values of the third and the eleventh overtone, i.e., δ*D* = Δ*D*3 − Δ*D*11, for the different measurements. To account for the sensor-specific differences in the Δ*D* traces during the initial growth regime, the δ*D* parameter was then normalized to its maximum value before the second injection. Figure 5 verifies that the time course of normalized δ*D* is similar for all measurements until the second injection at ca. 180 min, showing only minor variations in the time dependence due to the differences observed in initial bacterial adhesion and growth dynamics. Upon injection of phage-free medium (*E. coli* only control), δ*D* drops slightly in all traces but quickly starts to increase again, indicating resumed growth. At longer times, δ*D* saturates when growth is arrested. Here, the saturation value varies considerably between repeated measurements due to the different numbers of growing bacteria and microcolonies on the sensor surfaces. If the *Bacillus* phage phi29 is present in the injected medium, δ*D* shows a similar behavior with the saturation level within the range of variation observed for the phage-free measurements. In contrast, injection of *E. coli* phage T7 results in a larger drop in δ*D* by about 80%, which remarkably is independent of the T7 concentration (compare red, green, and blue curves in Fig. 5a). For the high T7 concentration (red curves), δ*D* remains at this low value for the rest of the experiments. For the lower concentrations (green and blue curves), however, it slowly increases again due to the resumed growth of the surviving bacteria. Nevertheless, addition of phi29 (violet curve) shows a similar behavior as *E. coli* in the absence of any phages (black curves).

To further illustrate and quantify the differences between the normalized δ*D* curves with and without T7 phages, we have calculated the second derivative of each curve for the period after the final flushing with phage-free medium. The resulting derivative curves allow a clear distinction between the two conditions, as the experiments with phages produce curves with mostly positive values, whereas the phage-free curves have mostly negative values (see Fig. S14a). Therefore, the values of both the sum and the mean of each derivative curve can be used to discern phage infection and lysis (see Fig. S14b, c). As a more direct measure, however, the δ*D* value at a selected timepoint after the final flushing can be utilized as well. At timepoint 240 min, i.e., after the drop in signal due to phage injection has saturated, δ*D* has a significantly smaller value in the presence (+ T7) than in the absence (− T7) of infectious phages (Fig. 5b).

## Conclusion

In summary, we have verified the potential of QCM-D to detect bacteriophage infections of surface-adhering bacteria in real time using T7 infection of *E. coli* as a model system. For ease of use in practical screening campaigns, we have not employed any chemical modifications of the sensors but immobilized the *E. coli* cells directly on the bare gold surface. The absence of any surface modifications also promotes reuse of the sensors in multiple experiments, as organic residues and cell debris can be removed using the standard cleaning procedure described in the “Materials and methods” section. Bacterial adsorption, adhesion, and growth at the sensor surface result in complex and overtone-dependent behaviors of the Δ*f* and Δ*D* traces. Various alterations in these behaviors are observed during phage infection and especially upon subsequent lysis of the infected bacterial cells, which are distinctly different from purely non-specific adsorption of non-infectious phages on the bacteria and the exposed sensor surface. This clearly demonstrates the ability of QCM-D to detect and monitor phage infection and lysis of bacterial cells.

By comparing sensor responses to infectious and non-infectious phages, we identified a single measurement parameter, i.e., the difference between the third and the eleventh overtones of the Δ*D* trace, δ*D* = Δ*D*3 − Δ*D*11, which enables a clear distinction between lytic phage infection and non-specific phage adsorption. Most importantly, when normalized to the maximum value observed in the first bacterial growth phase, this parameter appears more robust with regard to sensor-specific differences than the original Δ*D* traces, enabling a more reliable identification of phage-induced bacterial lysis. Using this parameter δ*D*, the complete T7 phage infection process could be followed within 4 h, including the formation of the *E. coli* films on the sensor surfaces. However, it should be stressed that T7 represents an ideal case with a very short lytic cycle of only 17 min. For other phages with longer lytic cycles, unambiguous detection of cell lysis will take considerably longer. Future experiments may optimize the experimental conditions and minimize the time to detect each phage-host combination individually. Nevertheless, the combination of multichannel QCM-D setups with automated liquid-handling systems may enable the efficient screening of medium-sized phage collections against target pathogens. Finally, since QCM-D is also able to monitor the removal of biofilms from the sensor surface in real time [48, 49], the presented approach may be further employed to screen phages being active also against bacterial biofilms.

## Supplementary Information

Below is the link to the electronic supplementary material.

Supplementary file1 (PDF 1.75 MB)

## Data Availability

The datasets generated during the current study are available in the Zenodo repository, 10.5281/zenodo.14515122.
