# Supplementary material for: Monitoring phage infection and lysis of surface-immobilized bacteria by QCM-D
Source: Anal Bioanal Chem. 2025 Feb 25;417(10):2143–53. doi: 10.1007/s00216-025-05803-5 (PMC11961536; doi:10.1007/s00216-025-05803-5)
Supplement: Supplementary file 1 — Supplementary file1 (PDF 1.75 MB) [file 216_2025_5803_MOESM1_ESM.pdf]

Supplementary Information

Monitoring phage infection and lysis of surface-immobilized bacteria by QCM-D

Bhanu K. Pothineni,[1] René Probst,[1] Dorothee Kiefer,[2] Verena Dobretzberger,[3] Ivan Barišić,[3] Guido Grundmeier,[1] and Adrian Keller[1,*]

[1] *Technical and Macromolecular Chemistry, Paderborn University, Warburger Str. 100, 33098 Paderborn, Germany*

[2] *Institute of Biology, University of Hohenheim, 190h, Garbenstr. 30, 70599 Stuttgart, Germany*

[3] *Molecular Diagnostics, Center for Health and Bioresources, AIT Austrian Institute of Technology GmbH, 1210 Vienna, Austria*

*E-mail: adrian.keller@uni-paderborn.de

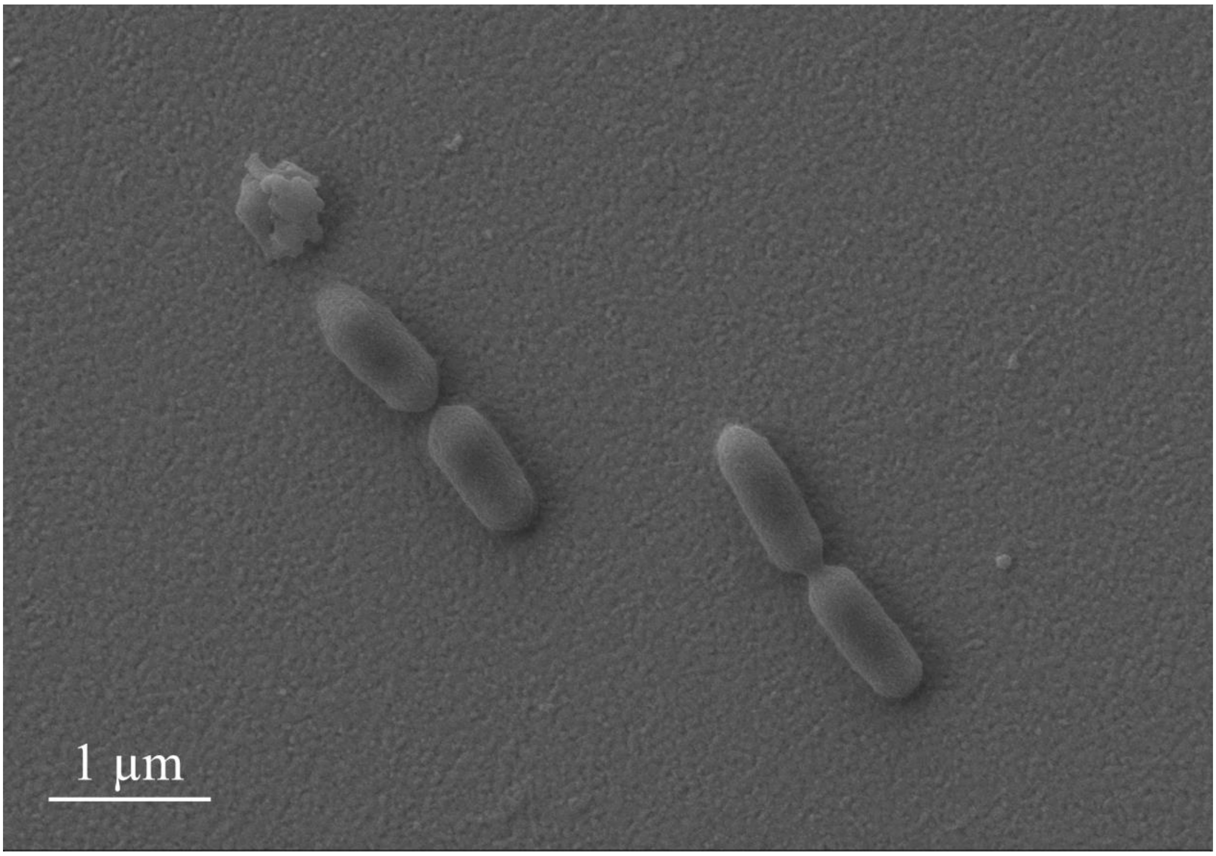

Figure S1: SEM image representation of a typical bacterial cell division.

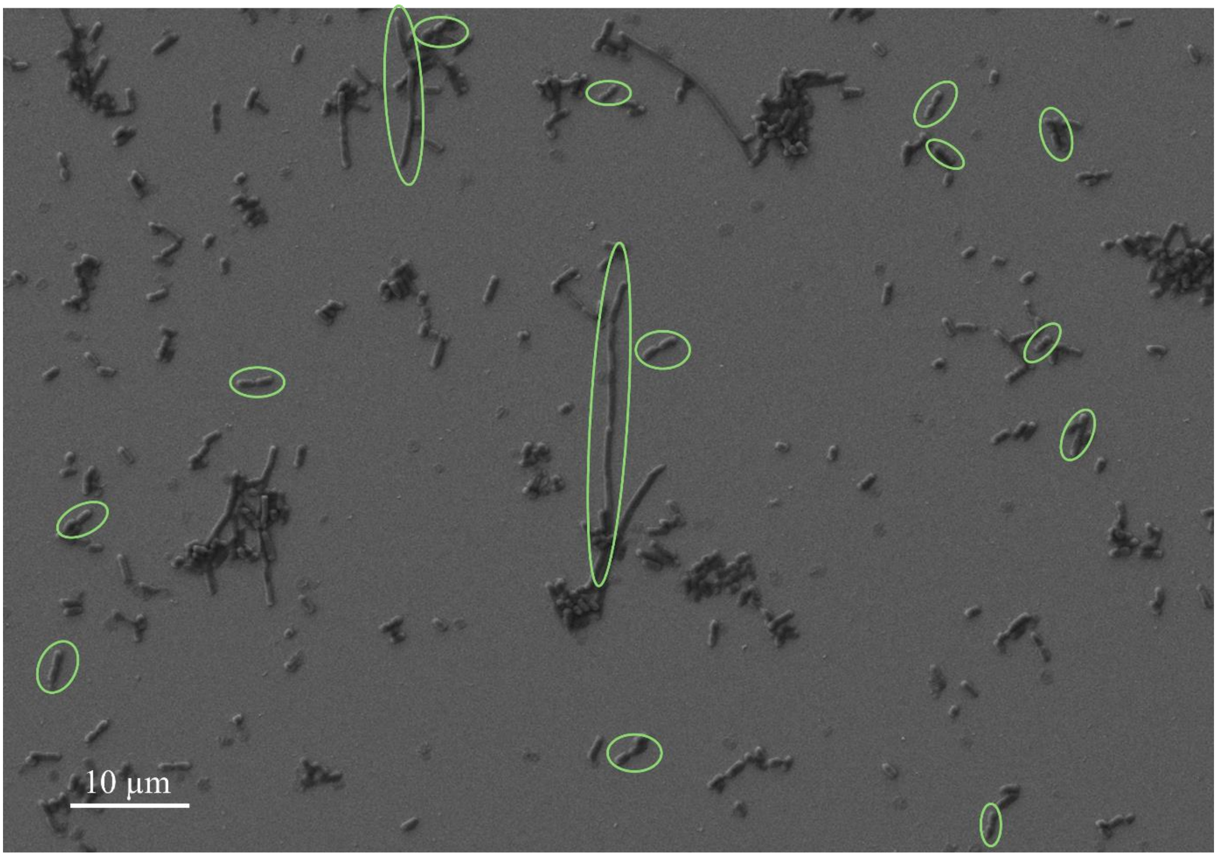

Figure S2: SEM image of the sensor surface after the experiment in Figure 1 without phages. Dividing bacteria with and without notable cell separation are highlighted.

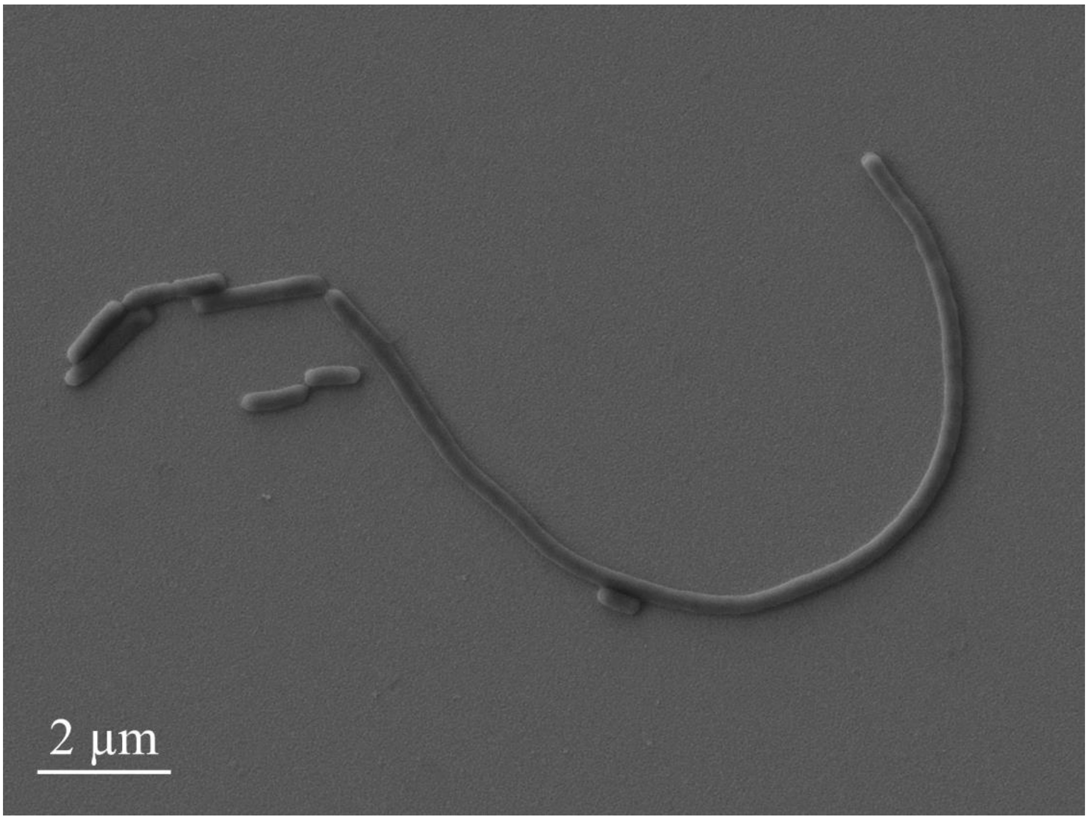

Figure S3: Zoomed SEM image of bacteria growing to lengths of several microns without cell division.

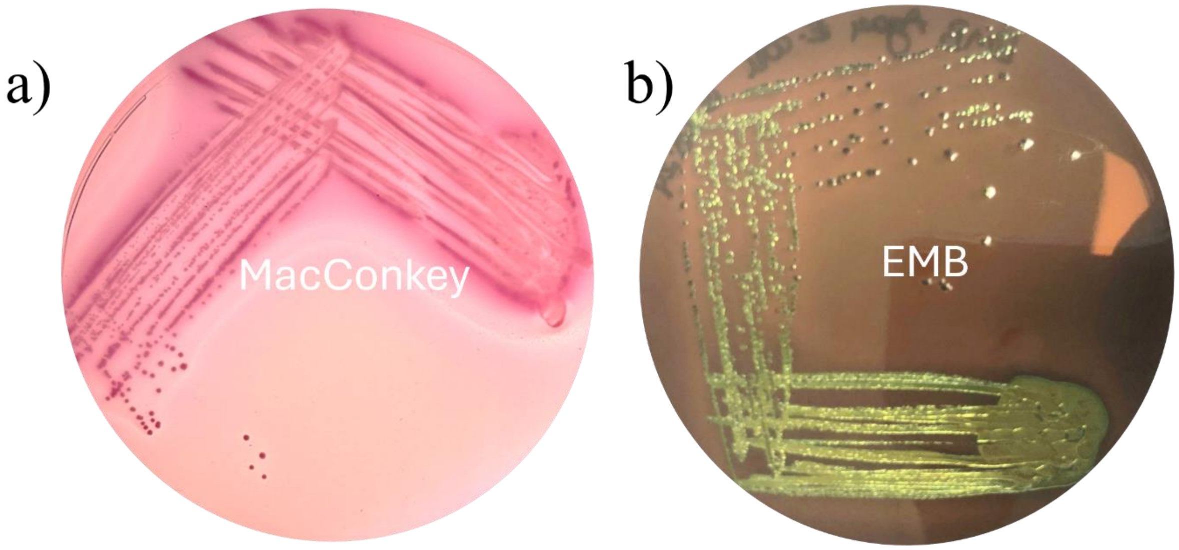

Figure S4: a) *E. coli* quadrant streaked on MacConkey agar showing pink non-mucoid colonies of lactose-fermenting *E. coli*. b) *E. coli* quadrant streaked on EMB agar showing colonies with green metallic sheen.

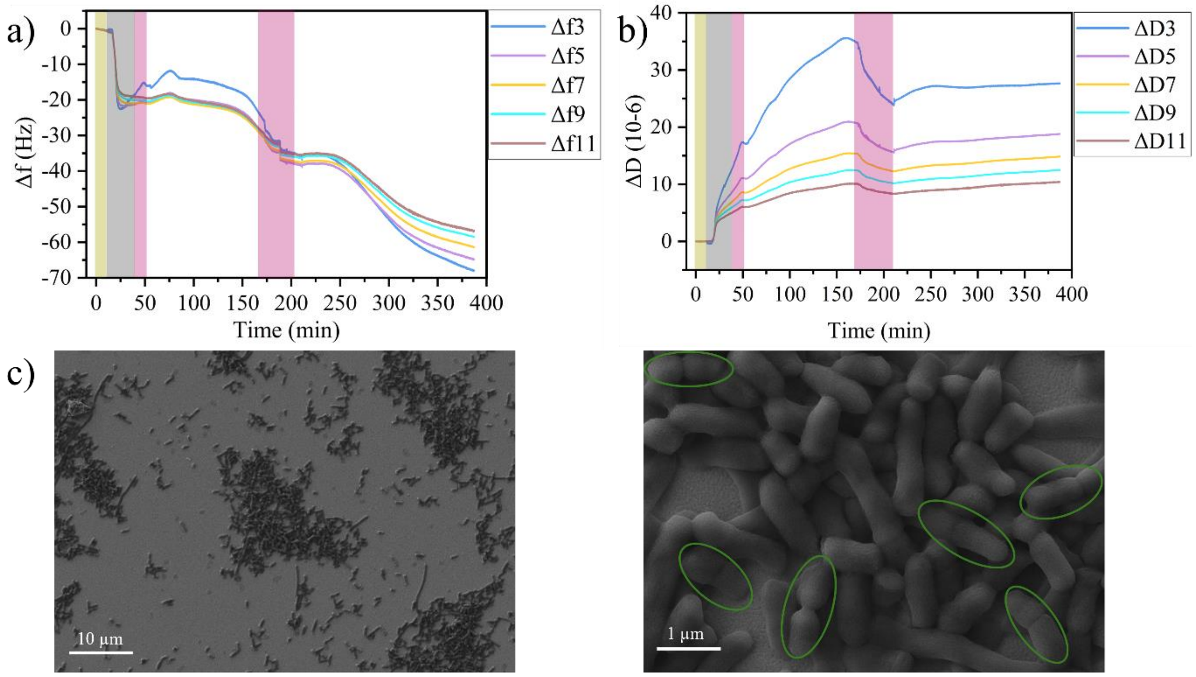

Figure S5: Replicate of the data shown in Figure 1. Change in frequency Δf (a) and dissipation ΔD (b) during *E. coli* adsorption and growth on the QCM-D sensor surface. Shaded regions indicate the injection of different media: yellow – PBS, grey – bacteria in medium, pink – medium. c) SEM images of the sensor surface after the experiment at two different magnifications. Dividing bacteria are highlighted.

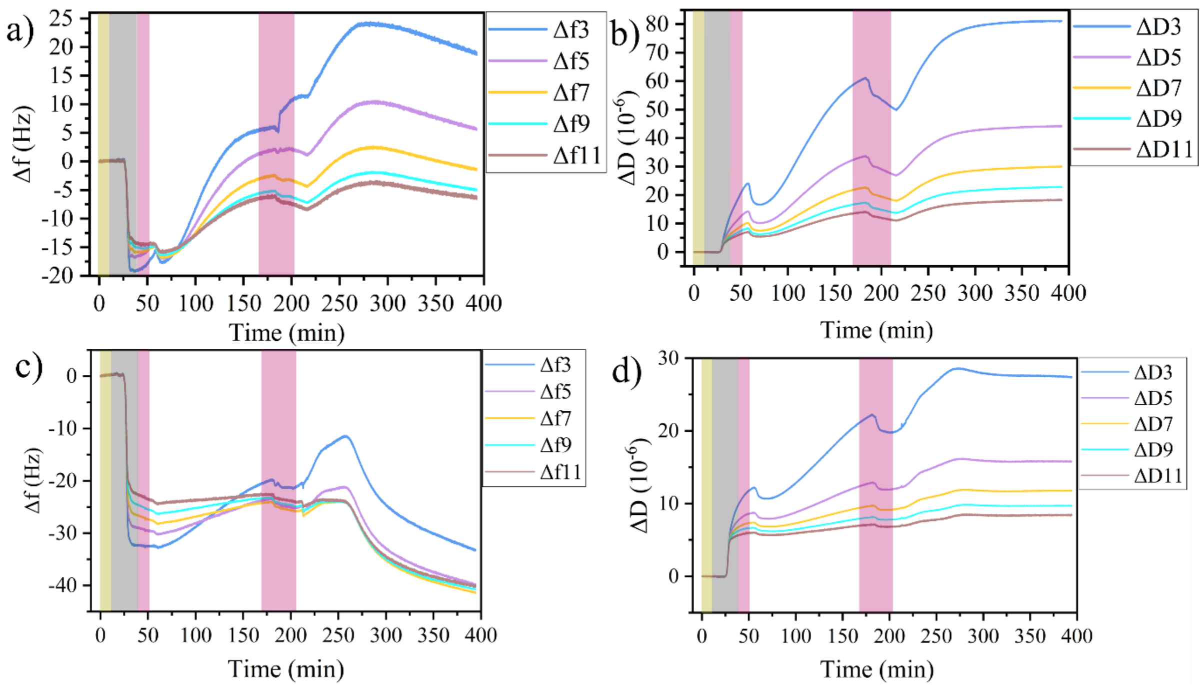

Figure S6: Replicates of the data shown in Figure 1. Change in frequency Δf (a,c) and dissipation ΔD (b,c) during *E. coli* adsorption and growth on the QCM-D sensor surface. Shaded regions indicate the injection of different media: yellow – PBS, grey – bacteria in medium, pink – medium.

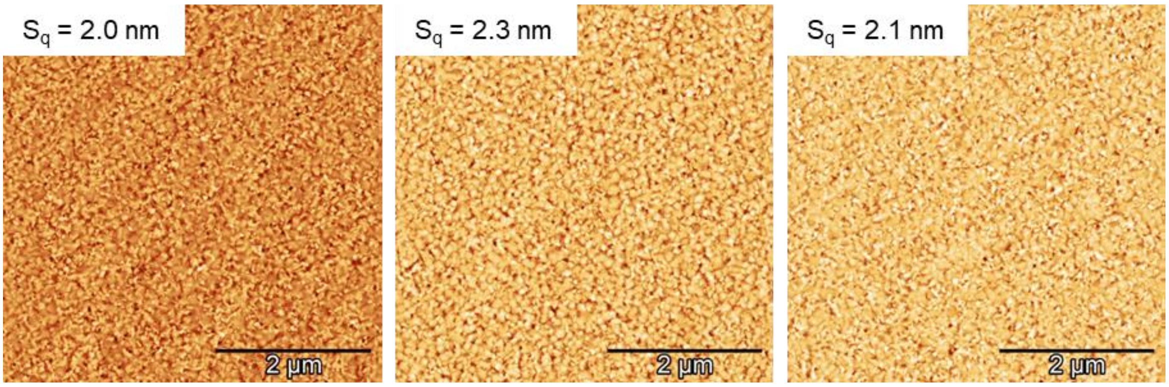

Figure S7: AFM images of the gold electrode surfaces of three QCM-D sensors from different batches. Height scales are 20 nm. The values of the root-mean-square (RMS) surface roughness S_q are given in the images.

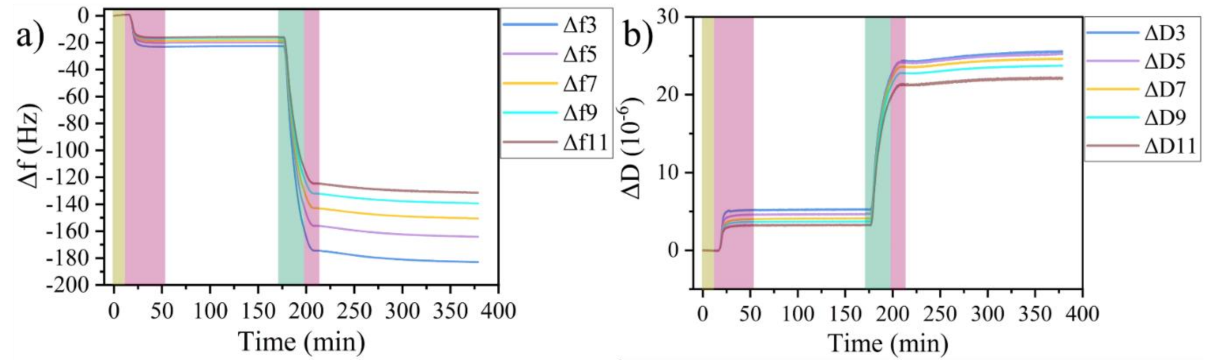

Figure S8: Change in frequency Δf (a) and dissipation ΔD (b) during T7 adsorption on the QCM-D sensor surface. Shaded regions indicate the injection of different media: yellow – PBS, pink – medium, green – T7 phages in medium (8 x 10^7 PFU/mL).

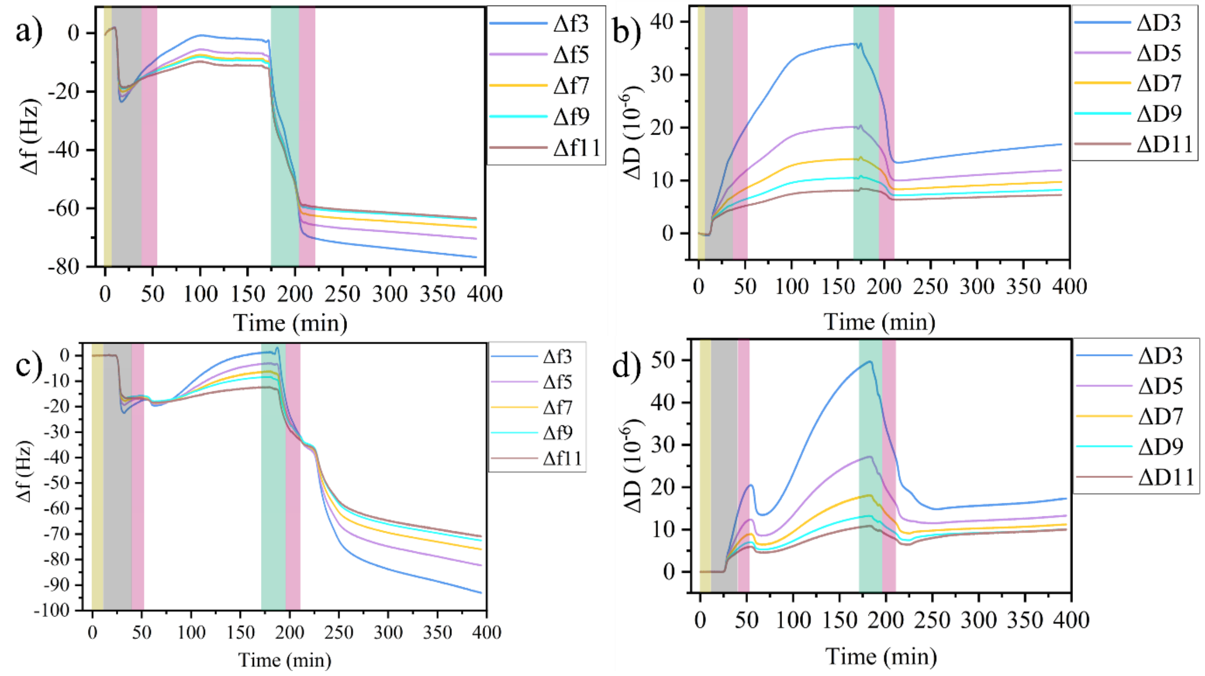

Figure S9: Replicates of the data shown in Figure 2. Change in frequency Δf (a,c) and dissipation ΔD (b,c) during *E. coli* adsorption, growth, and T7 infection on the QCM-D sensor surface. Shaded regions indicate the injection of different media: yellow – PBS, grey – bacteria in medium, pink – medium, green – T7 phages in medium (8 x 10^7 PFU/mL).

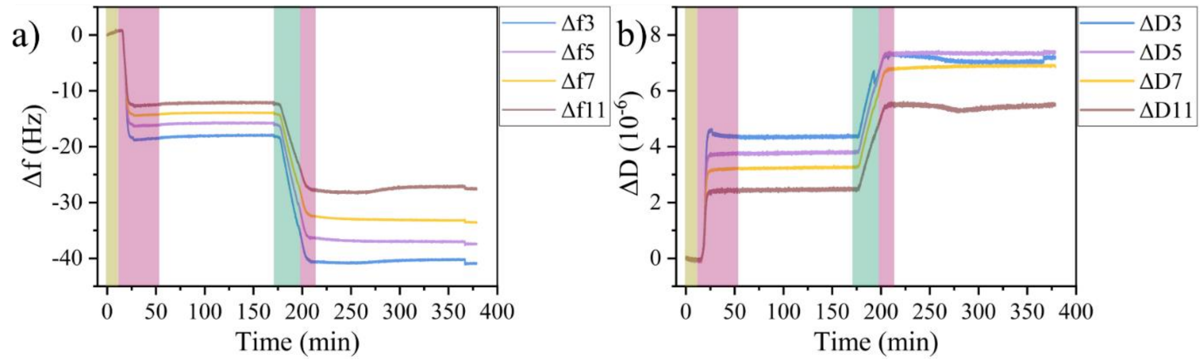

Figure S10: Change in frequency Δf (a) and dissipation ΔD (b) during T7 adsorption on the QCM-D sensor surface. Shaded regions indicate the injection of different media: yellow – PBS, pink – medium, green – T7 phages in medium (2 x 10^6 PFU/mL).

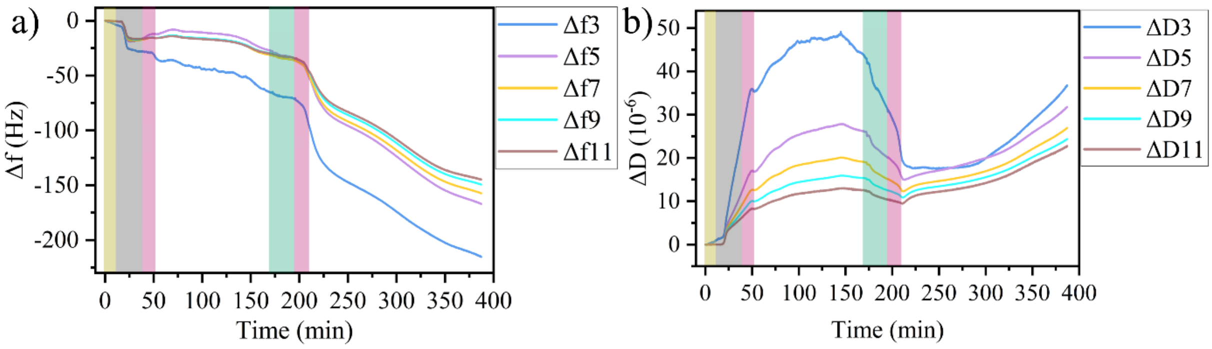

Figure S11: Change in frequency Δf (a) and dissipation ΔD (b) during *E. coli* adsorption, growth, and T7 infection on the QCM-D sensor surface. Shaded regions indicate the injection of different media: yellow – PBS, grey – bacteria in medium, pink – medium, green – T7 phages in medium (4 x 10^5 PFU/mL).

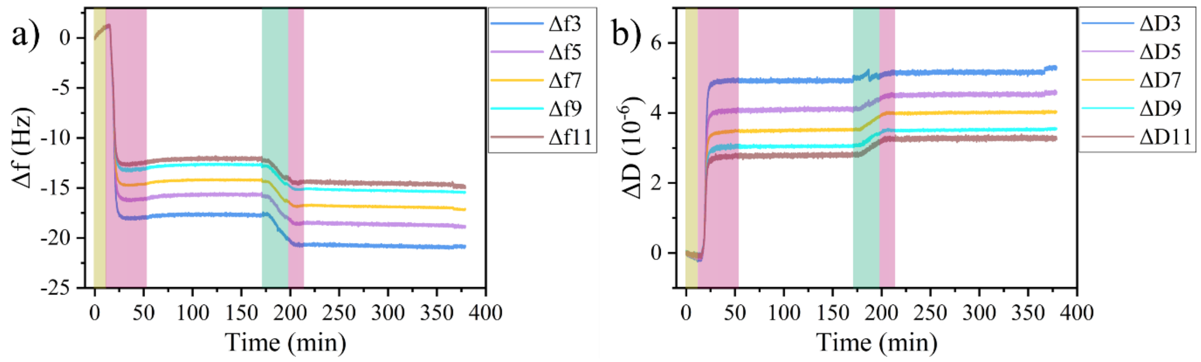

Figure S12: Change in frequency Δf (a) and dissipation ΔD (b) during T7 adsorption on the QCM-D sensor surface. Shaded regions indicate the injection of different media and buffer: yellow – PBS, pink – medium, green – T7 phages in medium (4 x 10^5 PFU/mL).

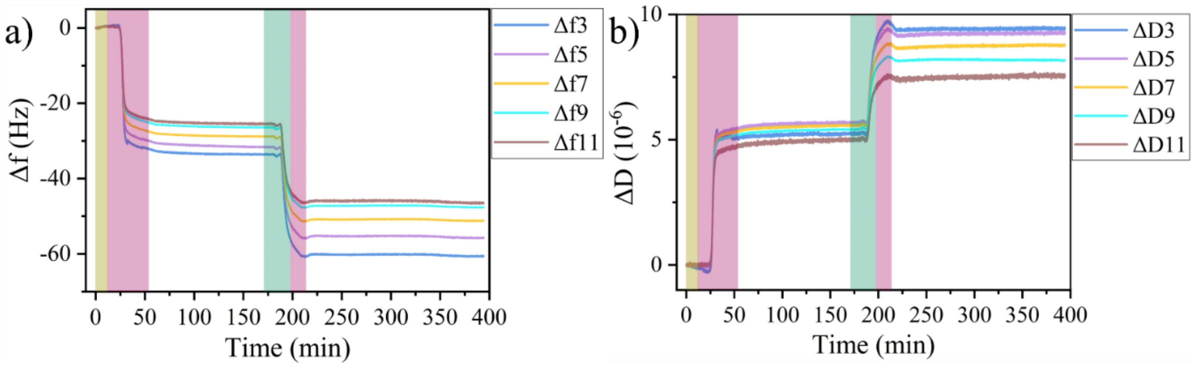

Figure S13: Change in frequency Δf (a) and dissipation ΔD (b) during phi29 adsorption on the QCM-D sensor surface. Shaded regions indicate the injection of different media and buffer: yellow – PBS, pink – medium, green – phi29 phages in medium (2 x 10^8 PFU/mL).

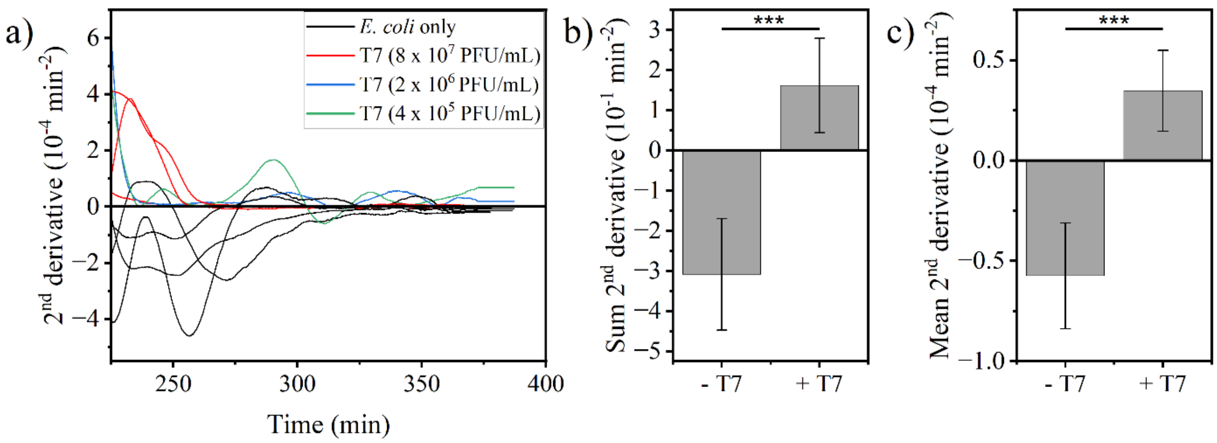

Figure S14: a) Second derivative of the normalized δD traces shown in Figure 5a after the final flushing with phage-free medium. b,c) Comparison of the sum (b) and the mean values (c) of the curves shown in a) averaged over all *E. coli* only experiments (-T7) and T7 experiments (all concentrations, +T7). Values are given as the mean ± standard deviation. Statistical significance was determined by two-tailed t-test and is indicated as *** ($p < 0.001$).
